# Intention to use electronic medical record and its predictors among health care providers at referral hospitals, north-West Ethiopia, 2019: using unified theory of acceptance and use technology 2(UTAUT2) model

**DOI:** 10.1186/s12911-020-01222-x

**Published:** 2020-09-03

**Authors:** Mohammedjud Hassen Ahmed, Adina Demissie Bogale, Binyam Tilahun, Mulugeta Hayelom Kalayou, Jorn Klein, Shegaw Anagaw Mengiste, Berhanu Fikadie Endehabtu

**Affiliations:** 1 Department of Health Informatics, Institute of Public Health, Mettu University, P.o.box: 196, Metu Zuria, Ethiopia; 2 Department of Health Informatics, Institute of Public Health, University of Gondar, Gondar, Ethiopia; 3 University of South-Eastern Norway, Post office box 235, N-3603 Kongsberg, Norway

**Keywords:** Electronic medical record system, Intention to use, UTAUT2model, Ethiopia

## Abstract

**Background:**

Electronic Medical Records (EMRs) are systems to store patient information like medical histories, test results, and medications electronically. It helps to give quality service by improving data handling and communication in healthcare setting. EMR implementation in developing countries is increasing exponentially. But, only few of them are successfully implemented. Intention to use EMRs by health care provider is crucial for successful implementation and adoption of EMRs. However, intention of health care providers to use EMR in Ethiopia is unknown.

**Objective:**

The aim of this study was to assess health care provider’s intention to use and its predictors towards Electronic Medical Record systems at three referral hospitals in north-west, Ethiopia, 2019.

**Methods:**

Institutional based cross-sectional explanatory study design was conducted from March to September among 420 health care providers working at three referral hospitals in north-west Ethiopia. Data were analyzed using structural equation model (SEM). Simple and multiple SEM were used to assess the determinants of health care providers intention to use EMRs. Critical ratio and standardized coefficients were used to measure the association of dependent and independent variables, 95% confidence intervals and *P*-value were calculated to evaluate statistical significance. Qualitative data was analyzed using thematic analysis.

**Result:**

The mean age of the study subjects was 32.4 years ±8.3 SD. More than two-third 293(69.8%) of the participants were male. Among 420 health care providers, only 167 (39.8%) were scored above the mean of intention to use EMRs. Factors positively associated with intention to use EMRs were performance expectancy (β = 0.39, *p* < 0.001), effort expectancy (β = 0.24,*p* < 0.001),social influence (β = 0.18,*p* < 0.001),facilitating condition (β = 0.23,*p* < 0.001), and computer literacy (β = 0.08,*p* < 0.001). Performance expectancy was highly associated with intention to use EMRs.

**Conclusion:**

Generally, about 40 % of health care providers were scored above the mean of intention to use EMRs. Performance expectancy played a major role in determining health care providers’ intention to use EMRs. The intention of health care providers to use EMRs was attributed by social influence, facilitating condition in the organization, effort expectancy, performance expectancy and computer literacy. Therefore, identifying necessary prerequisites before the actual implementation of EMRs will help to improve the implementation status.

## Background

In previous decades, healthcare sectors in many countries have experienced a number of changes due to advancement of information and communication technology (ICT) [1]. Health Information Technology (HIT) is a broad term that describes the technology and infrastructure used to record, analyze, and disseminate population health data [2, 3]. Integrating different information technologies (ITs) into the healthcare system of both developed and developing countries is not all about modernizing the health system but increase patient safety, improves communication, supports practicing evidence based decision and incorporating e-learning to remote health professionals. IT can used as a medium to access recent healthcare information, data handling and processing activities among health care providers [4]. Electronic Medical Records (EMRs) is a system that store patient information like medical histories, test results, and medications electronically [5]. The most significant contribution of EMR is that a patient will have one electronic chart that can be accessed any time with in one organization.

With the increasing demand for digital information in health care, the Electronic Medical Records (EMRs) represent the main foundation of health information technology in health care setting. Implementation of various technologies like EMRs contributes in improving health care organizations workflow. Utilization of EMRs are important for both patients and health care professionals to retrieve medical history, treatment results and previous diagnosis [6]. Poor health information system implementation has been identified as a major challenge in the health-care system in many developing countries including sub-Saharan African countries [7]. Correct and up-to-date information is critical for the provision of high-quality clinical care, for clinical and health service research, planning and management of health systems [8]. Paper based record system has limitations on accuracy, timeliness, completeness, consistency and legibility of hand writing. As EMRs) have been taken into use in the clinical field, the patients’ data have become interoperable between different health care professionals in different service units and in different regions. The EMRs are expected to make a significant impact on health care data quality, healthcare outcomes and clinical practices [9–11].

Globally, many healthcare facilities tried to implement EMR system, mainly to improve health information recording process [12–16]. In the context of developing countries, several technological, organizational and social issues such as electrical power interruption, health professionals technology resistance, infrastructure and administrative problems have slowed the pace of implementation and adoption of EMRs [17]. Problems related to lack of planning, increased provider time, computer down time, lack of standards to interchange information, user resistance and threats to confidentiality negatively contribute for the successful implementation of EMR [4]. Even though there is a high expectation and interest in EMR as great prospect for improving quality, continuity, safety, and efficiency in healthcare, the overall adoption rate is relatively low [18, 19]. Fifty percent of health information system failed to utilized EMRs properly [6, 20, 21].

Intention to use is a measurement to which a person has formulated conscious plans to perform or not to perform some specified future behavior for using EMRs [17]. EMRs intention to use by healthcare providers is an essential requirement to ensure that the expected benefits will be materialized and to predict the actual utilization of EMRs [22]. Evidences revealed that health professionals acceptance to the new system and the potential disruptions and changes that follow is one of the major barrier to implement EMR [23–25]. So, identifying the necessary prerequisites before the actual implementation of EMRs will help to improve the implementation status.

In Ethiopia, the ministry of health in collaboration with Tulane University developed a comprehensive EMR software system called Smart Care. The system had been deployed in Diredawa, Bahirdar, Harar and Addis Ababa city administrations of Ethiopia as a pilot, and the ministry of health had also planned to scale it up to other hospitals and regions [7]. In its pilot implementation phase, health professionals’ resistance was reported to be the primary hindering factor to its successful adaptation. To the author’s’ knowledge, no study has been assessed intention to use EMRs among health care providers in Ethiopia. Therefore, this study aimed to determine intention of health care providers to use EMRs and associated determinants in selected referral hospitals in northwest Ethiopia.

### Theoretical model and hypotheses

The most notable model has been introduced as ‘Unified Theory of Acceptance and Use of Technology (UTAUT)’ [26] to explain the relation of independent and dependent variable. This model was extracted from eight previous theoretical models that includes theory of reasoned action (TRA), Social Cognitive Theory (SCT), Technology Acceptance Model (TAM), Theory of Planned Behavior (TPB), Motivational model, Model of PC utilization (MPCU), Combined TAM and TPB (C-TAM-TPB) and Innovation Diffusion Theory (IDT) [26, 27]. In 2012,unified theory of acceptance and use of technology developed to predict acceptance and use of technology [27] has been introduced. The aim of this model was to connect Unified Theory of Acceptance and Use of Technology (UTAUT). Considering the three key structures, and changing some relations of the original concepts of UTAUT and introducing the new relationships is called the Unified Theory of Acceptance and Use of Technology2 (UTAUT2). Therefore, in addition to the four common variables (performance expectancy, effort expectancy, social influence and facilitating conditions) with UTAUT, three key variables of hedonic motivation, price value and habit were added to UTAUT2 model as independent variables. The following are Hypothesis (H) of intention to use EMRs.

- (H1): Performance expectancy will positively influence health care providers towards intention to use EMRs
- (H2): Effort expectancy will positively influence health care providers towards intention to use EMRs
- (H3): Social influence will positively influence health care providers towards intention to use EMRs
- (H4): Facilitating condition will positively influence health care providers towards intention to use EMRs
- (H5): Hedonic motivation will positively influence health care providers towards intention to use EMRs
- (H6): Habit will positively influence health care providers towards intention to use EMRs
- (H7): Computer literacy will have positively influence on health care provider’s intention to use EMRs

**Fig. 1:**
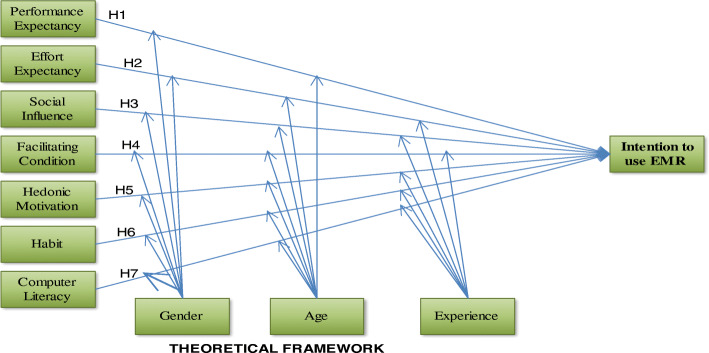
Theoretical framework of Adopted UTAUT2 modeling

This study considered seven independent constructs and one dependent construct. Price value was excluded from the study because of the study participants were not direct purchasers of the system. User behavior which was considered as dependent variable in the original UTAUT2 was not measured in this study due to the proposed technology is a predicted technology, which has not been implemented currently in Ethiopia and no current actual use of EMR in these three referral hospitals. The proposed model was not tested in Ethiopia context especially motivation, habit and computer literacy predictors. The effect of moderation on intention to use EMRs was not tested in previous studies. Finally, the proposed theoretical model is presented in Fig. 1.

## Methods

### Study design and setting

Institution based cross-sectional explanatory study design was conducted from March to September, 2019 at three referral hospitals in Amhara region, north-west Ethiopia. Amhara region consists of 10 administrative zones, one special zone and 78 urban centers. The regional health bureau is piloted EMRs in three pilot areas at Bahir-dar health center, Lalibela health center and Boru meda primary hospital. The region has planned to scale up to other facilities. This study was conducted at three selected referral hospitals namely; Felege-hiwot, University of Gondar, and Debre-Markos referral hospitals.

### Study participants and sample size determination

In this study, health care providers working at three hospitals were taken as the study population.

The sample size was calculated using single population proportion formula with the following assumptions: 95% confidence level (CI), Z (1-α/2) =1.96), an expected proportion of intention to use EMRs as 50, and 5% margin of error. Considering a 10% non-response rate, the minimum final sample size was 423. For the qualitative part; eleven study participants were participated and level of saturation used to determine the minimum sample size.

### Sampling procedure

Study participants were selected using population proportion allocation from the three referral hospitals namely; university of Gondar, Felege-hiwot and Debre-markosl. Participant from each hospital were selected using simple random sampling technique. Purposive sampling method was used to select participants for the qualitative part.

### Data collection

A structured self-administered questionnaire was prepared based on Unified Theory of Acceptance and Use technology2 (UTAUT2) [27, 28]. The questionnaire comprised of socio demographic and intention to use EMRs variables. It was prepared in English language. The tool was pre-tested on a group of 22 healthcare providers who did not belong to the study hospitals. Six data collectors and three supervisors participated in the data collection process. Two days training were given to the data collectors and supervisors on the objective of the study and data collection procedures. The principal investigator and supervisors did a daily supportive supervision on data collectors, and data back-up were performed to prevent data loss. For qualitative data, in-depth interview was conducted and open ended questions were used. The minimum and maximum times for in-depth interview were 34 and 47 min respectively.

### Study procedure

Study participants were selected from three referral hospitals found in North-West, Ethiopia. Among the three referral hospitals from University of Gondar, Felege-hiwot and Debre-markos referral hospital, study participants for quantitative data were selected using population proportion allocation for each referral hospital. Simple random sampling technique was used to select study participants from the three referral hospital. In-depth interview was used for the qualitative part.

### Data management and analysis

Epi-Info version 7 for data entry, and SPSS version 20 and AMOS version 23 were used to analyze data. Descriptive statistics was computed to describe socio demographic characteristics and univariate to assess magnitude of intention to use EMRs. Simple and multiple SEM analysis were carried out to identify the most important variables which could determine intention to use EMRs in the study areas. Chi-square, Goodness of Fit Index (GFI), Adjusted Goodness of Fit Index (AGFI), Root Mean Square Error of Approximation (RMSEA) and Normal Fit Index (NFI) were used for checking fitness of the model. For the structural equation model, path coefficients were interpreted as SEM coefficients with the critical ratio calculated using bootstrapping. Similar to linear regression analysis, R^2^ represents the proportion of variance in the endogenous constructs which can be explained by the predictors. Critical Ratio and standardized path coefficients were used to measure the association of dependent and independent variables, 95% confidence intervals and *P*-value were used to evaluate statistical significance (*p*-value< 0.05). Qualitative data was analyzed using thematic analysis manually.

### Reliability and validity of the research

Cronbach’s alpha reliability coefficients were computed to determine the internal consistency of all research constructs: Cronbach’s alpha of 0.7 or above indicates high reliability, between 0.5–0.7 indicates moderate reliability and less than 0.5 indicates low reliability. Performance expectancy (α = 0.70), effort expectancy (α = 0.75), facilitating condition (α = 0.69), computer literacy (α = 0.77) and habit (α = 0.80) were assessed by four Likert item questions and social influence (α = 0.65), hedonic motivation (α = 0.86) and intention to use EMRs (α = 0.71) were assessed by three item questions (Table 1).

**Table 1:** Reliability of predictors and intention to use EMRs among health care providers in Northwest, Ethiopia

Construct	Sample size	Number of items	Cronbach’s Alpha
Performance Expectancy	420	4	0.70
Effort Expectancy	420	4	0.75
Social Influence	420	3	0.65
Facilitating Condition	420	4	0.69
Hedonic motivation	420	3	0.86
Computer Literacy	420	4	0.77
Habit	420	4	0.80
intention to use EMRs	420	3	0.71

**Table 2:** Socio-demographic characteristics of health care providers at three referral hospitals in north-west Ethiopia, 2019

Variables	Frequency	Percent
**Gender**		
Male	127	30.2%
Female	293	69.8%
**Age in years**		
20–29	233	55.5%
30–39	91	21.7%
40–49	72	17.1
50–59	24	5.7
**Profession**		
Nurse	141	33.6%
Psychiatry	7	1.7%
Optometry	8	1.9%
Midwifery	85	20.2%
Physician	126	30.0%
Health officer	4	1.0%
Anesthesia	7	1.7%
Laboratory	11	2.6%
Radiology	8	1.9%
Physiotherapy	2	0.5%
Pharmacy	16	3.8%
Other	5	1.2%
**Working experience in years**		
1–3	185	44.0%
3–5	61	14.5%
5–10	114	27.1%
> 10	60	14.3%

## Results

Out of the 423 questionnaires sent to the respondents, 420 were responded with a response rate of 99.30%. The mean age of the study participants’ was 32.4 years ±8.3 SD. More than two-third 293(69.8%) of the participants were male. One third 141(33.6%) of the study participants were Nurses, 126(30.0%) were physicians and only 2(0.5%) were physiotherapist. About 185(44.0%) of the study participants had less than 3 years working experience, and only 60 (14.3%) had more than 10 years working experience (Table 2).

Intention to use EMRs among health care providers was assessed by three Likert items having reliability test of Cronbach alpha (α = 0.71). From “I intend to use EMRs system in the future” questions, Hundred sixty five of respondents (39.3%) agree that they were intended to intention to use EMRs in the future (Table 3).

**Table 3:** Intention to use EMRs among healthcare providers at three referral hospitals in northwest Ethiopia

Items	Strongly disagree	Disagree	Neutral	Agree	Strongly agree
I intend to use the EMRs system in the future	10(2.4%)	101(24.0%)	96(22.9%)	165(39.3%)	48(11.4%)
I predict I will use the EMRs system in the future	6(1.4%)	90(21.4%)	123(29.3%)	159(37.9%)	42(10.0%)
I plan to use the EMR system in the future	5(1.2%)	92(21.9%)	108(25.7%)	159(37.9%)	56(13.3%)

### Mean score of all predictors and intention to use EMRs in UTAUT2 model

Performance expectancy, effort expectancy, facilitating condition, computer literacy and habit measured using four Likert item questions had a mean score of 14.1 (SD ± 2.9), 14.8(SD ± 2.9), 13.3(SD ± 3.4), 12.4(SD ± 3.4) and 13.6(SD ± 3.7) respectively and social influence, hedonic motivation and intention to use EMRs also measured using three Likert questions and had a mean score of 11.0 (SD ± 2.2), 10.4 (SD ± 3.4) and 10.1 (SD ± 2.4) respectively.

### Magnitude of intention to use EMRs

In this study, about 167(39.8% [95.0%: CI 35.0–45.0) of the study participants scored above the mean. The mean score of intention to use EMR was 10.1 with standard deviation of 2.4. The maximum and minimum scores were 15 and 3 respectively.

### Correlation analysis between predictors and intention to use EMRs

There is positive correlation between performance expectancy (PE) (*r* = 0.83,*p* < 0.001), facilitating condition (FC) (*r* = 0.76,*p* < 0.001),effort expectancy (EE) (*r* = 0.73,*p* < 0.001),social influence (SI) *(r* = 0.71,*p* < 0.001) and computer literacy (CL) (*r* = 0.36,*p* < 0.001) with intention to use EMRs among health care providers.

### Simple structural equation model analysis

**Fig. 2:**
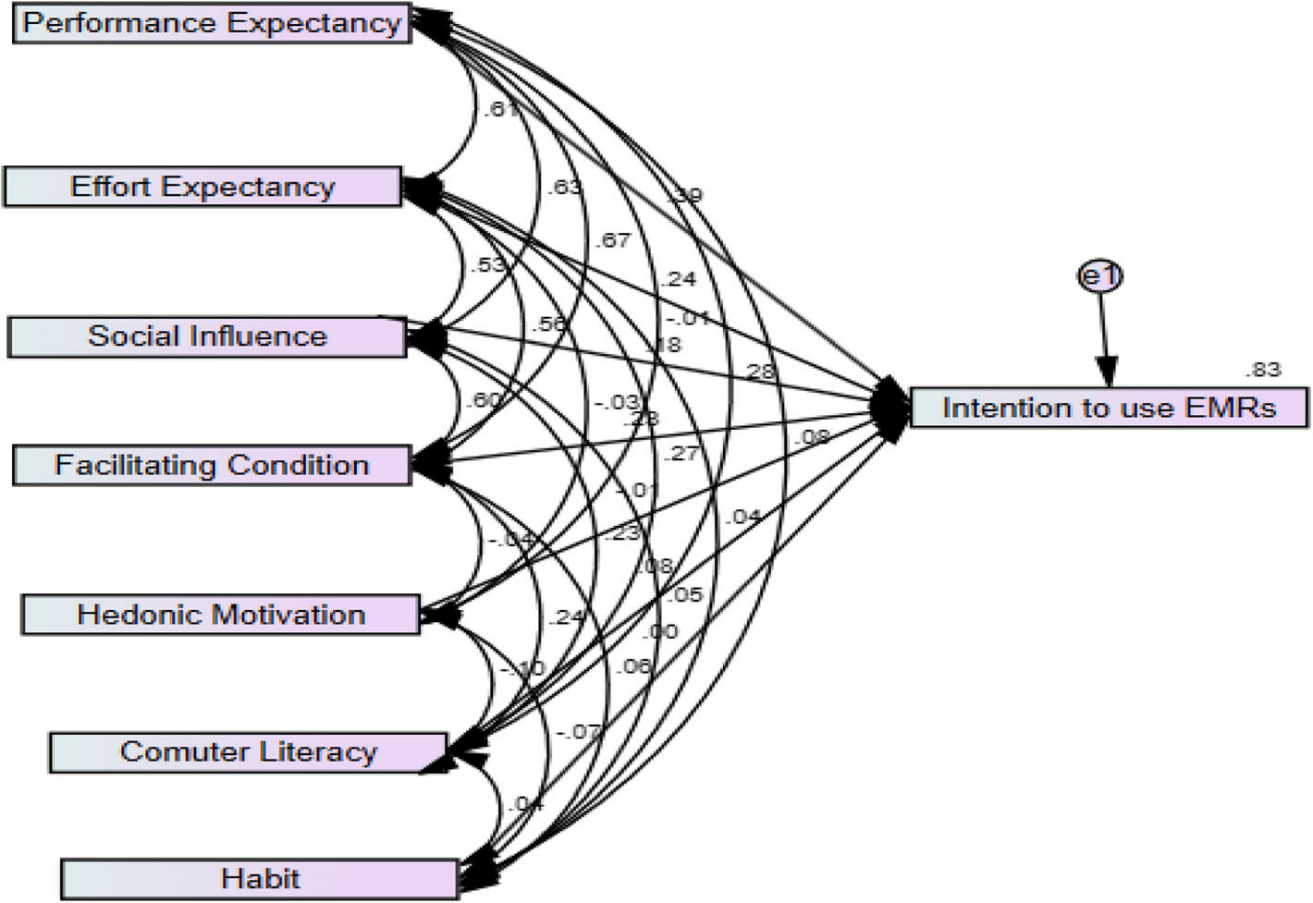
Predictors and intention to use EMR among health care providers at referral hospitals North-west Ethiopia, 2019

Figure 2 depicts the structural equation model showing path coefficients and R^2^. The Bootstrap method was used to assess the statistical significance of the path coefficients of predictors. With respect to the key determinants of intention to use EMRs, performance expectancy has the most direct influence on intention to use EMRs, followed by effort expectancy, facilitating conditions, social influence and computer literacy. All values having *p*-value less than 0.2 in simple SEM were entered in to multiple SEM. Standardized coefficient and R^2^ values were used to interpret the effects and variability in the dependent variable respectively. The independent variables explained 83.0% of dependent variable. Significance independent predictors were discussed using 95.0% Confidence Interval (CI) and *p*-value less than 0.05 was taken as a cut off point for association between independent variables and dependent variable.

As shown in Fig. 2, performance expectancy (the degree to which a user believes that EMR will improve performance) has a positive effect on intention to use EMR (β = 0.39, *p* < 0.01). Similarly, the degree of ease of use, effort expectancy (H2) associated with system use has a positive influence on intention to use EMR (β = 0.24 *p* < 0.01). Social influence (H3); the degree to which a user perceives the importance of others’ opinion with respect to EMR use also plays a significant role in intention to use an EMR(β = 0.18 *p* < 0.01). The degree to which an individual believes that an organizational and technical infrastructure exists to support system use facilitating conditions (H4) is also significant with respect to intention to use EMRs (β = 0.23 *p* < 0.01). Finally, computer literacy (H6) has a significant influence on intention to use EMRs (β = 0.08 *p* < 0.01). Hedonic motivation (H5) (β = − 0.01 *p* > 0.05) and habit (H7) (β = 0.01 *p* > 0.05) has no significance influence on intention to use EMRs. Overall, 83.0% of the variance with respect to intention to use EMRs was reasonably explained by the constructs of predictors (Table 4).

**Table 4:** Multiple Structural equations modeling association between predictors and intention to use EMRs among health care providers in Northwest, Ethiopia, 2019

			Estimate	Standard Error (SE)	Critical Ratio (CR)	P	95% confidence interval	
							Lowerr	Upper
PE	➔	IU	0.39	0.03	12.47	*	0.29	0.49
EE	➔	IU	0.24	0.02	9.04	*	0.17	0.33
SI	➔	IU	0.18	0.03	6.48	*	0.12	0.24
FC	➔	IU	0.23	0.02	7.90	*	0.16	0.30
HM	➔	IU	−0.01	0.02	−0.56	0.57	−0.05	0.03
CL	➔	IU	0.08	0.02	3.96	*	0.03	0.13
HB	➔	IU	0.01	0.01	0.13	0.90	−0.04	0.04

Dependent variable: IU, * significance at *P* < 0.05

Note: *PE* Performance expectance, *EE* Effort expectancy, *SI* Social Influence, *FC* Facilitating Condition, *HM* Hedonic Motivation, *CL* Computer Literacy, *HB* Habit, *IU* Intention to Use

### Testing potential moderators: gender, age and experience

In this section, possible moderations of the variables Gender, Age and Experience on the relationship of each of the significant independent variable (PE, EE, SI, FC and CL) with the dependent variable (IU) were investigated. For each independent variable and for each potential moderator, two SEM were conducted. The first SEM includes the dependent variable on one of the independent variable (simple slopes). In the second SEM, both independent variable and an interaction term (independent variable * moderator) were added to AMOS to control for the moderator. If both the interaction term and significance difference between interaction term and independent variable of slopes were found to be significant (C.R difference between estimates of Moderator variable> 1.96 or C.R < -1.96) and *p*-value less than 0.05), then the proposed moderator variable was confirmed as a Moderator.

### Moderating effect of gender among health care providers on intention to use EMRs

As shown in the Table 5, there was no statistically significance difference between being male or female for all predictors on intention to use EMRs. The detail is presented in Table 5.

**Table 5:** Moderating effect of gender among health care providers at referral hospitals in north-west Ethiopia, 2019

Variables		Std. Coefficient	C.R	*P*–value	Critical Ratio difference between Estimates of Gender
Performance Expectancy	Female	0.82	15.9	*	C.R = 0.38 and *p* = 0.71 (Not Supported)
	Male	0.84	25.9	*	
Effort Expectancy	Female	0.80	14.9	*	C.R = -1.6 and *p* = 0.1 (Not Supported)
	Male	0.70	16.9	*	
Social influence	Female	0.71	11.5	*	C.R = -0.2 and *p* = 0.96 (Not Supported)
	Male	0.72	17.6	*	
Facilitating Condition	Female	0.76	13.0	*	C.R = -1.5 and *p* = 0.98 (Not Supported)
	Male	0.76	19.9	*	
Computer Literacy	Female	0.40	4.9	*	C.R = -0.6 and *p* = 0.33 (Not Supported)
	Male	0.34	6.1	*	

Dependent variable: IU, * significance at *P* < 0.05

The relation between performance expectancy and intention to use EMRs is negatively moderated by Age (β = − 0.5, *p* = 0.01) but other moderation variable of predictor’s had no influence on intention to use EMRs. The detail is presented in Table 6.

**Table 6:** Moderating effect of age and experience among health care providers at referral hospitals in north-west Ethiopia, 2019

Predictors	Interaction	Std. coefficient	C.R	*P*-value	Significance difference b/n interaction term with predictor	Confirmation
Performance Expectancy (PE)	PE_X_Age	−0.50	−2.8	0.01	Critical Ratio = 17.3and *P* = 0.00*	Supported
Effort Expectancy (EE)	EE_X_Age	−0.14	−0.6	0.52		(Not Supported)
	EE_X_Experience	−0.03	−0.2	0.87		(Not Supported)
Social Influence (SI)	SI_X_Age	−0.36	−1.6	0.11		(Not Supported)
	SI_X_Experience	−0.33	− 1.7	0.08		(Not Supported)
Facilitating Condition (FC)	FC_X_Age	−0.27	−1.5	0.14		(Not Supported)
	FC_X_Experience	−0.15	−1.1	0.26		(Not Supported)
Computer Literacy (CL)	CL_X_Age	−0.11	0.4	0.69		(Not Supported)
	CL_X_Experience	−0.08	−0.4	0.70		(Not Supported)

Dependent variable: IU, *significance at *P* < 0.05

### SEM model fit and assumptions

The table below (Table 7) presents the various SEM model fit indices (chi-square, GFI, AGFI, CFI, RMSEA and NFI) with their theoretical cut-offs. The 6 statistics values indicate that the SEM fit is fulfilled the assumption. SEM is based on a set of assumptions: The relationship between the dependent variable and each of the predictor should be linear (the expected value of the error terms is equal to zero); the error terms are normally distributed and errors have equal variances regardless of the values of the independent variables (homoscedastic assumption). Therefore, all fit indices support that the developed structural model fits well with the data. Based on the above information, the model passes SEM assumption.

**Table 7:** SEM fitness for intention to use EMRs among health care providers in north-west, Ethiopia, 2019

Fit indices	Threshold Value	Authors	Results obtained	Conclusion
Chi-square	≤3	Bentler (1990).	1.06	Accepted
Goodness-of-fit-index (GFI)	> 0.9	Chau (1997)	0.99	Accepted
Adjusted goodness-of-fit-index (AGFI)	> 0.8	Chau (1997)	0.98	Accepted
Comparative fit index (CFI)	> 0.9	Bentler (1990)	1.0	Accepted
Root mean square error of approximation (RMSEA)	< 0.05	Byrne (2001)	0.01	Accepted
Normed fit index (NFI)	> 0.9	Bentler & Bonett (1980)	0.99	Accepted

### Result of qualitative data

Exploring intention to use EMRs using qualitative method was used to find out barriers and challenges of using EMRs. Majority of the participants agreed that they are intended to use EMRs when necessary prerequisites to use EMRs full field in their organization like training and infrastructures as well as generator. One participant expressed his opinions about intention to use EMR in the following ways. *“I am very eager to use EMR because it facilitates my tasks and easily to store and retrieve patient information using key words. So, I am looking forward to use it (EMRs)” 21–30 year’s old. Another participant said “I am not that much good in computer skill but I have seen the important of EMRs for all parties and if I get at least two more training days about it (EMRs),I will be definitely delighted to use in this hospital if implemented fully”* 21–30 year’s old.

Few of the participants raised their concerns regarding using EMRs and they were negatively intended to use EMRs in the future. Their concern is that it is not appropriate using computer in front of the patient. It will also create double burden to use both EMRs and paper based on health workers during power off. One participant said the following: “*I will have double burden during power off when our hospital implements EMRs fully because I have to write in the paper and, when light is on, I will expect another task converting it (hard copy) into EMR which is disgusting if there is no generator”* 21–30 year’s old*.*

Generally, there were many factors that hindered intention to use of EMRs in selected referral hospitals. Shortage of financial incentives and priorities, poor electricity supply and internet connectivity, and primary user’s limited computer skills were the hindering factors mentioned by participants. Financial supports and training of primary users were identified as facilitator to use EMRs. Majority of participants expected EMRs to improve their performance, easy to use and will have enough computer capacity about using EMRs in the future. Most participants were recommended that awareness needs to be given for those health care providers, who have negative intention towards using EMRs. For example one participant said *“EMRs help health professional in different ways like it saves time to jot down notes, helps to see the drug adverse reaction for the patient in which it is difficult in today’s system and generally promotes effectiveness and efficiency in health care industry*” 31–40 year’s old.

## Discussion

This study examined to assess intention to use EMRs and to identify predictors among health care providers at referral hospitals in North-West Ethiopia using UTAUT2 model. Performance expectancy, effort expectancy, social influence, facilitating condition and computer literacy were predictors statistically associated with intention to use EMRs.

This study revealed that magnitude of intention to use EMRs was assessed by three Likert items with reliability test of Cronbach’s alpha (α = 0.71). In this study, about 167(39.8% [95.0%: CI 35.0–45.0) of the study participants scored above the mean. Score of intention to use EMRs. The mean score of intention to use EMRs was 10.1 with standard deviation of ±2.40. The expected maximum and minimum score was 15 and 3 respectively.

This study also identified predictors that influence intention to use EMRs. This study showed that performance expectancy played an important role in predicting health care providers intention to use EMRs (β =0.39, *p* < 0.001). This finding is supported by different literatures. A cross sectional study conducted in Ethiopia (*P* < 0.001) [29], South Dakota(β = 0.27,*p* < 0.01) [30],Indonesia(β = 0.30,*p* < 0.05) [31],Canada(β = 0.35*,p* < 0.001) [32],Taiwan(β = 0.29,*p* < 0.001) [33], south Korea (β =0.30, *p* < 0.05) [34] and Jordan(β = 0.39,*p* < 0.01) [35]) positively influence intention to use EMRs among health care providers. This study revealed that EMR systems are directly related to improvements in the performances of healthcare providers. This might be due to People’s intention to use EMRs in the work place are influenced by usefulness of EMRs for improving patient safety, and enhancing daily workflow [36].

This study also found that effort expectancy was statistically significant predictor of intention to use EMRs among health care providers (β =0.24, *p* < 0.001). This finding is in line with a study done in Ethiopia (*p* < 0.005) [29], Kenya (β = 0.18, *p* < 0.001) [37] and comparative study done in US and Portugal (β = 0.16; *P* < 0.01) [38], US (β = .187, *p* < .01) [39] positively influence intention to use EMRs. This might be due to individuals perception of users often want to be faced with a system that is easy to use provided that the system can meet the needs of their practical applications [40]. This might be also due to their perception that using EMRs will simplify their tasks and, information and data can also be managed in a clear and systematic way.

Another significance predictor of intention to use EMRs is social influence (β = 0.18, *p* < 0.001). Social influence positively affects intention to use EMRs This result is consistent with a study done in morocco (B = 3.29, *p* < 0.001) [41], Taiwan (β = 0.16, *p* = 0.015) [38], South Korea (β = 0.10, *p* < 0.05) [34] and America (β_=_0.198 *p* < 0.05) [42]. Health care providers might perceive that external pressure towards using a new system could be possibly from the hospital administration, patients or health professionals [43]. So, healthcare staff members need to perceive pressure from external body, which is important for them to increase their motivation and intention to use EMRs in their organization. Mechanisms that encourage role modeling and peer support, such as champions and super users, might increase health care provider’s acceptance of EMRs.

In this study, facilitating condition played an important role in predicting health care providers intention to use EMRs (β = 0.23, *p* < 0.001). This is in line with a study conducted in North Central Nigeria (β = 0.11, *p* < 0.05) [44],South Africa (β = 0.28, *p* < 0.05) [45],Malaysia (β = 0.16,*p* < 0.05) [46] and Ontario Canada (β = .11, *p* < .01) [32]. It could be due to the fact that health care providers believe that the resources and technical support should be available to use the EMR in their workplace [43]. As training and organization readiness is the main part of facilitating condition, health care providers might also perceive that they will get favorable condition to use EMRs in the future if they take training.

Another significant predictor of the current study was computer literacy. Computer literacy was positively associated with intention to use EMRs (β = 0.08, *p* ≤ 0.001). This finding is supported by a study conducted in Malaysia (β = 0.92 *p* ≤ 0.01) [46], Taiwan (β = 0.14, *p* = 0.009) [47] and Iran(*R* = 0.40, *P* < 0.01) [1]. This might be due to that those who had computer literates believe that they will not face difficulty if EMRs system will be implemented in the future.

The finding of this study revealed that the influence of performance expectancy on health care provider’s intention to use EMRs was negatively moderated by Age (β = − 0.5, *p* ≤ 0.01). This finding is in line with other study conducted in China (β = − 0.33, *p* < 0.001) [48]. A probable reason for this could be that older health care providers have less exposure for information technology. In contrast, due to their greater interaction with technologies growing up and in medical school, younger health care providers are more likely to feel more comfortable and able to adopt and use EMR system.

## Conclusion

In conclusion, about 40 % of health care providers were scored above the mean of intention to use EMRs. Factors associated with intention to use EMRs were performance expectancy, effort expectancy, social influence, facilitating condition and computer literacy influence intention to use EMRs. Among the five influencing factors, performance expectancy had more significant prediction power of intention to use EMRs than other predictor variables. The intention of health care providers to use EMRs was attributed by social influence, facilitating condition in the organization, effort expectancy, performance expectancy and computer literacy. Therefore, identifying necessary prerequisites before the actual implementation of EMRs will help to improve the implementation status.

## Data Availability

The datasets generated and/or analyzed during the current study will be available upon reasonable request from the corresponding author.

## Abbreviations

ADFI: Adjusted goodness of fit index
CFI: Comparative fit index
CI: Confidence interval
CR: Critical ratio
EE: Effort expectancy
EFMOH: Ethiopian federal ministry of health
E-HEALTH: Electronic health
EHR: Electronic health record
EMR: Electronic medical records
FC: Facilitating condition
GFI: Goodness of fit index
HIT: Health information technician
ICT: Information communication technology
IT: Information technology
IU: Intention to use
NFI: Normed fit index
NGO: Non-governmental organization
PE: Performance expectancy
RMSEA: Root mean square error of approximation
SD: Standard deviation
SEM: Structural equation modeling
SI: Social influence
SPSS: Statistical package for social science
TAM: Technology acceptance model
UTAUT: Unified theory of acceptance and use technology
WHO: World health organization
